# Synthesis and Antitumor Activity of 5-Bromo-7-azaindolin-2-one Derivatives Containing a 2,4-Dimethyl-1*H*-pyrrole-3-carboxamide Moiety

**DOI:** 10.3390/molecules21121674

**Published:** 2016-12-06

**Authors:** Jun Zhang, Weiyi Shen, Xiaoning Li, Yun Chai, Senjun Li, Kai Lv, Huiyuan Guo, Mingliang Liu

**Affiliations:** 1 Institute of Medicinal Biotechnology, Chinese Academy of Medical Sciences and Peking Union Medical College, Beijing 100050, China; jun0215@126.com (J.Z.); lxn18273645@163.com (X.L.); lvkailk@hotmail.com (K.L.); imbhyguo@126.com (H.G.); 2 Zhejiang Starry Pharmaceutical Co. Ltd., Xianju 317300, China; swy109@starrypharma.com (W.S.); lisj0966@163.com (S.L.)

**Keywords:** 5-bromo-7-azaindolin-2-ones, synthesis, antitumor activity

## Abstract

We report herein the design and synthesis of a series of novel 5-bromo-7-azaindolin-2-one derivatives containing a 2,4-dimethyl-1*H*-pyrrole-3-carboxamide moiety. These newly synthesized derivatives were evaluated for in vitro activity against selected cancer cell lines by MTT assay. Results revealed that some compounds exhibit broad-spectrum antitumor potency, and the most active compound **23p** (IC_50_: 2.357–3.012 μM) was found more potent than Sunitinib (IC_50_: 31.594–49.036 μM) against HepG2, A549 and Skov-3, respectively.

## 1. Introduction

**Figure 1:**
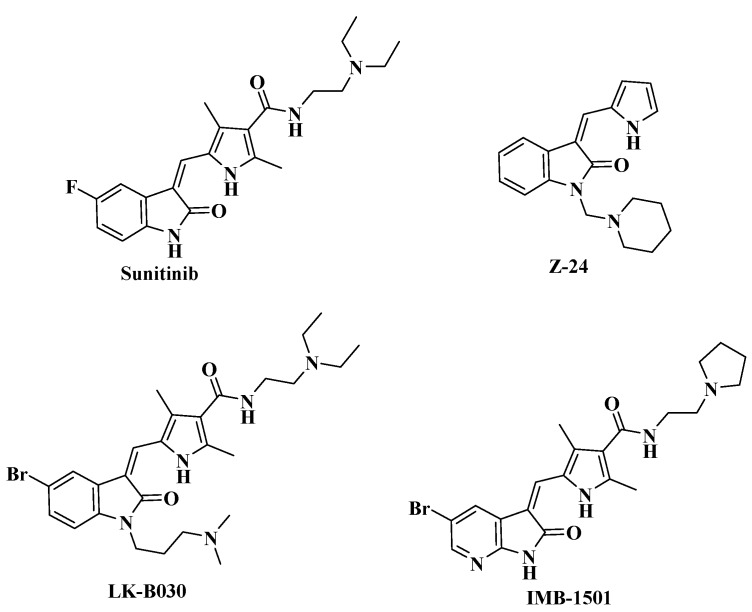
Structures of Sunitinib, Z-24, LK-B030 and IMB-1501.

Sunitinib (Figure 1) is a new multitargeted oral anti-angiogenic and antitumor drug that has been recently approved against gastrointestinal stromal tumors (GIST) and advanced renal cell carcinoma (RCC) [1]. It is in clinical studies for the treatment of other solid tumors, such as pancreatic neuroendocrine tumors [2], meningioma [3], metastatic breast cancer [4] and non-small cell lung cancer [5].

Recently, structural modifications mainly at the 3- and 5-positions of the indolin-2-one ring of Sunitinib have made considerable progress in the ability to increase antitumor activity through inhibition on different receptors [6,7,8]. As early lead compounds discovered in our lab, Z24 and LK-B030 (Figure 1) bearing a (piperidin-1-yl)methyl and a (3-dimethylamino)propyl group at the N-1 position, respectively, display a broad spectrum of antitumor activity by inhibiting angiogenesis in new blood vessels [9,10,11]. More recently, we reported a series of novel 5-halogenated-7-azaindolin-2-one derivatives and found IMB-1501 to have better in vitro activity than Sunitinib against the entire tested cancer cell lines [12].

As part of our continuing modifications on Sunitinib as a potential antitumor drug candidate, we planned to explore other possibilities for diversification of the 2-(pyrrolidin-1-yl)ethyl group and the linker flexibility on the amide bond. Thus, a series of novel 5-bromo-7-azaindolin-2-one derivatives containing a 2,4-dimethyl-1*H*-pyrrole-3-carboxamide moiety were designed, synthesized and evaluated for their antitumor activity in this study. Our primary objective was to optimize the potency of these compounds against a set of solid tumors and contribute to the development of new antitumor agents. A preliminary structure-activity relationship (SAR) study is also explored to facilitate the further development of 5-bromo-7-azaindolin-2-ones.

## 2. Results and Discussion

**Scheme 1:**
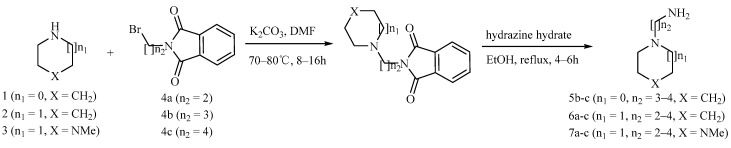
Synthesis of amine derivatives **5**–**7**.

**Scheme 2:**
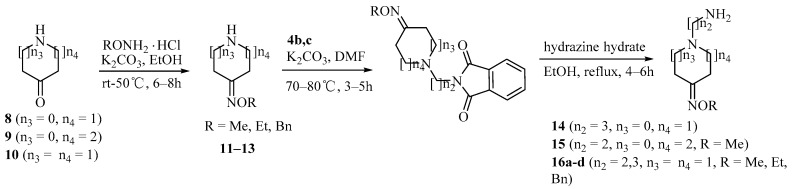
Synthesis of amine derivatives **14**–**16**.

**Scheme 3:**
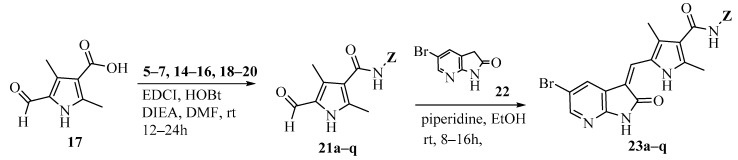
Synthesis of target compounds **23a**–**q**.

Detailed synthetic pathways to heterocyclic amine derivatives **5**–**7** and **14**–**16**, which are commercially unavailable, and target compounds **23a**–**q** are depicted in Scheme 1, Scheme 2 and Scheme 3, respectively. Amine derivatives containing aminoalkyl groups **5**–**7** were easily obtained from pyrrolidine **1**, piperidine **2** and *N*-methylpiperazine **3** by nucleophilic substitution with *N*-(2-bromoethyl)/*N*-(3-bromopropyl)/*N*-(4-bromobutyl)phthalimides **4a**–**c** in the presence of K_2_CO_3_ in DMF at 70–80 °C, respectively, followed by treatment of the resulting condensates with hydrazine hydrate in ethanol under reflux condition (Scheme 1).

Condensation of pyrrolidin-3-one **8**, piperidin-3-one **9** and piperidin-4-one **10** with *O*-alkylhydroxyamines gave compounds **11**–**13**. Amine derivatives **14**–**16** were prepared from oximes **11**–**13** by coupling with **4b**,**c** and hydrazinolysis, sequentially (Scheme 2).

Amidation of 5-formyl-2,4-dimethylpyrrole-3-carboxylic acid **17** with **5**–**7**, **14**–**16** and commercially available (*S*)-1-amino-3-morpholinopropan-2-ol (**18**), *N*^1^,*N*^1^-dimethylpropane-1,3-diamine (**19**) and *N*^1^,*N*^1^-diethylpropane-1,3-diamine (**20**) in the presence of 1-ethyl-3-(3-dimethylaminopropyl)carbodiimide hydrochloride (EDCI), *N*-hydroxybenzotriazole (HOBt) and Ethyldiisopropylamine (DIEA) yielded compounds **21a**–**q**. Aldol condensation of 5-formyl-2,4-dimethylpyrrole-3-carboxamides **21a**–**q** with 5-bromo-7-azaindolin-2-one **23** in the presence of piperidine gave the target compounds **23a**–**q** (Scheme 3) [10,11]. All of the new synthetic compounds were well characterized by ^1^H-NMR, ^13^C-NMR and MS. As expected, the pyrrole-2-methylidene geometry at the 3-position of the 7-azaindolin-2-one ring was confirmed to have the Z-configuration [12,13,14].

We first replaced the ethylidyne linker of Sunitinib with propylidyne or butylidyne, and the diethylamino group with a saturated heterocyclic amine (pyrrolidine, piperidine, piperazine, or hydroxylmorphine [15]) to synthesize the derivatives **23a**–**e**. For preliminary screening of antitumor candidates, the target compounds were investigated for cytotoxic activity in vitro against MCF-7 (a breast cancer cell line). It is encouraging that all of the initially designed molecules except **23e** exhibit higher inhibition (81.46%–87.29%) than Sunitinib (27.79%) at the concentration of 30 μM (Table 1). They were further evaluated for their in vitro antitumor activity in six human cancer cell lines, including MCF-7, HepG2 (liver carcinoma), HT-29 (colon adenocarcinoma), A549 (lung adenocarcinoma), PANC-1 (pancreatic carcinoma) and Skov-3 (ovarian carcinoma) by MTT assay [16].

The data reveal that 5-bromo-7-azaindolin-2-ones **23a**–**e** demonstrate increased activity (IC_50_: 3.103–65.054 μM) compared to Sunitinib (IC_50_: 29.257–65.606 μM) against all of the tested cancer cell lines (Table 1). In particular, compound **23c** exhibits a value of 3.103 μM against A549 and **23d** exhibits a IC_50_ value of 3.721 μM against Skov-3, which are 9.4- and 8.6-fold more potent than Sunitinib, respectively. It seems likely that the linker between the amide and the pyridine ring is well tolerated with an alkyl chain of C3/C4 (**23a**–**b**). Moreover, bearing a pyridine or piperidine or piperazine moiety with an alkyl chain of C2–4 is more favorable than the introduction of a hydroxy group on the alkyl linker (**23a**–**d** vs. **23e**).

Being encouraged by the above results, we further explored other possibilities for diversification of the linker or/and heterocyclic amine to design and synthesize the derivatives **23f**–**q** which were evaluated for their activity in selected cell lines HepG2, A549 and SKOV-3 (Table 2). When the ethylidyne linker (**23c**, **23d**) was replaced by a propylidyne or butylidyne moiety, the resulting compounds (**23f**, **23g**, **23i**) were found to have better activity (IC_50_: 5.023–7.803 μM) than Sunitinib (IC_50_: 31.594–49.036 μM). However, replacement of the butylidyne linker (**23i**) with a propylidyne moiety or opening of the piperidine ring (**23f**) led to decreased potency, although the corresponding compounds (**23h**, **23j**, **23k**) demonstrate similar activity.

Considering the importance of an oxime functional moiety of the C-7 side chain with respect to the antibacterial and/or antitumor activity of quinolones [17,18,19], the impact of an alkoxyimino group on the pyrrolidine or piperidine ring was also investigated. It is clear that the introduction of a methoxyimino group on the pyrrolidine ring is detrimental (**23l**). For the piperidine ring, the nature and position of the oxime group and linker greatly influence activity. For instance, the presence of a methoxyimino group at the para-position is more favorable than the meta-position (**23n** vs. **23m**). In addition, the propylidyne linker (**23o**) displays MIC values of 6.828–7.747 μM, which are 2.2- to 4.7-fold more potent than the ethylidyne linker (**23n**). It is also shown that the methyl group of the oxime moiety (**23o**) could be replaced by an ethyl (**23p**) or a benzyl (**23q**) one without obviously affecting the antitumor potency. Among the three, the most active compound **23p** (IC_50_: 2.357–3.012 μM) was found to be 11.3- to 18.4-fold more potent than Sunitinib against all of the tested cell lines, respectively (Table 2).

## 3. Experimental Section

Melting points were determined in open capillaries and are uncorrected. ^1^H-NMR and ^13^C-NMR spectra were determined on a Varian Inova-500 spectrometer (Varian, Inc., Palo Alto, CA, USA) in DMSO-*d*_6_ using tetramethylsilane as an internal standard. Electrospray ionization (ESI) mass spectra were obtained on an MDSSCIEX Q-Tap mass spectrometer (AB Sciex, Redwood City, MA, USA) and Advion Mass Express 2.1.243 (Advion BioSciences, Inc., Ithaca, NY, USA). The reagents were all of analytical grade or chemically pure. TLC was performed on silica gel plates (Merck, ART5554 60F254, Kenilworth, NJ, USA).

A mixture of heterocyclic amines (**1**–**3**, 5.9 mmol), 2-(2-bromoethyl/propyl/butyl)isoindoline-1,3-diones (**4a**–**c**, 7.0 mmol) and potassium carbonate (7.0 mmol) in *N*,*N*-dimethylformamide (10 mL) was stirred for 8–16 h at 70–80 °C. Then the reaction mixture was cooled to temperature and was added water. The aqueous layer was extracted with dichloromethane (30 mL × 3) and the combined organic layer was concentrated under reduced pressure. The residue was solved in ethanol (10 mL) and treated with 80% hydrazine hydrate (10.5 mmol). The reaction mixture was stirred at reflux for 4–6 h and concentrated under reduced pressure to give the title compounds **5b**–**c**, **6a**–**c** and **7a**–**c** as yellow oils or off-white solids.

A mixture of pyrrolidin-3-one/piperidin-3-one/piperidin-4-ones (**8**–**10**, 15.0 mmol), *O*-alkylhydroxyl amine hydrochlorides (18.0 mmol) and potassium carbonate (30.0 mmol) in ethanol (50 mL) was stirred for 6–8 h at 25–50 °C. Then the mixture was cooled to temperature and filtered, the filter cake washed with ethanol. The filtrate was concentrated under reduced pressure to afford the oximes **11**–**13** as oils. Amine derivatives **14**, **15** and **16a**–**d** were prepared from the oximes **11**–**13** by coupling with **4b**,**c** and hydrazinolysis sequentially as described above.

A mixture of 5-formyl-2,4-dimethyl-1*H*-pyrrole-3-carboxylic acid (**17**, 3.0 mmol), 1-ethyl-3-(3-dimethylaminopropyl)carbodiimide hydrochloride (EDCI) (4.5 mmol) and HOBt (4.5 mmol) in *N*,*N*-Dimethylformamide (10 mL) was stirred for 0.5 h at room temperature. Then compounds **5**–**7**, **14**–**16** or **18**–**20** (4.5 mmol) and DIEA (6.6 mmol) were added. The reaction mixture was stirred for 12–24 h and later water was added. The aqueous layer was extracted with dichloromethane/methanol (10:1, 30 mL × 2) and the combined organic layer was concentrated under reduced pressure. A solution of the residue and 5-bromo-1,3-dihydro-2*H*-pyrrolo[2,3-*b*]pyridin-2-one (**22**, 3.0 mmol) in ethanol (10 mL) was stirred at temperature for 8–16 h. The precipitate was filtered, purified via silica gel column chromatography (methanol/dichloromethane 40:1) and recrystallized from ethanol to afford the title compounds **23a**–**q** (26%–33%, two steps) as yellow solids.

*(Z)-5-[(5-Bromo-2-oxo-1,2-dihydro-3H-pyrrolo[2,3-b]pyridin-3-ylidene)methyl]-2,4-dimethyl-N-[3-(pyrrolidin-1-yl)propyl]-1H-pyrrole-3-carboxamide* (**23a**): Yield: 26%. m.p.: 222–224 °C. ^1^H-NMR (500 MHz, DMSO-*d*_6_) δ: 13.44 (s, 1H), 11.63 (s, 1H), 8.47 (d, *J =* 2.0 Hz, 1H), 8.10 (d, *J =* 2.0 Hz, 1H), 7.85 (s, 1H), 7.74 (t, *J =* 5.5 Hz, 1H), 3.26 (q, *J =* 12.5 Hz, 2H), 2.46–2.47 (m, 6H), 2.44 (s, 3H), 2.42 (s, 3H), 1.65–1.71 (m, 6H) ppm. ^13^C-NMR (400 MHz, DMSO-*d*_6_) δ: 169.1, 164.4, 151.1, 144.2, 137.8, 131.8, 127.7, 126.7, 126.0, 122.2, 121.4, 112.5, 111.0, 53.5, 53.3, 52.0, 37.2, 28.0, 23.0, 13.4, 10.5, 7.2. MS-ESI (*m*/*z*): 472.6 (M + H)^+^, 474.6 (M + H)^+^.

*(Z)-5-[(5-Bromo-2-oxo-1,2-dihydro-3H-pyrrolo[2,3-b]pyridin-3-ylidene)methyl]-2,4-dimethyl-N-[4-(pyrrolidin-1-yl)butyl]-1H-pyrrole-3-carboxamide* (**23b**): Yield: 28%. m.p.: 230–232 °C. ^1^H-NMR (500 MHz, DMSO-*d*_6_) δ: 13.44 (s, 1H), 11.63 (s, 1H), 8.47 (d, *J =* 2.0 Hz, 1H), 8.10 (d, *J =* 2.0 Hz, 1H), 7.85 (s, 1H), 7.74 (t, *J =* 5.5 Hz, 1H), 3.22 (q, *J =* 12.5 Hz, 2H), 2.43 (s, 3H), 2.41 (s, 3H), 2.37–2.40 (m, 6H), 1.65–1.67 (m, 4H), 1.48–1.53 (m, 4H) ppm. ^13^C-NMR (400 MHz, DMSO-*d*_6_) δ: 169.1, 164.3, 151.1, 144.1, 137.6, 131.8, 127.7, 126.8, 126.0, 122.3, 121.8, 112.5, 110.9, 55.4, 53.6, 38.6, 27.4, 26.0, 23.0, 13.3, 10.5. MS-ESI (*m*/*z*): 486.6 (M + H)^+^, 488.6 (M + H)^+^.

*(Z)-5-[(5-Bromo-2-oxo-1,2-dihydro-3H-pyrrolo[2,3-b]pyridin-3-ylidene)methyl]-2,4-dimethyl-N-[2-(piperidin-1-yl)ethyl]-1H-pyrrole-3-carboxamide* (**23c**): Yield: 28%. m.p.: 239–241 °C. ^1^H-NMR (500 MHz, DMSO-*d*_6_) δ: 13.45 (s,1H), 11.63 (s, 1H), 8.48 (d, *J =* 2.0 Hz, 1H), 8.10 (d, *J =* 2.0 Hz, 1H), 7.86 (s, 1H), 7.52 (t, *J =* 5.5 Hz, 1H), 3.34 (t, *J =* 6.5 Hz, 2H), 2.46 (s, 3H), 2.44–2.45 (m, 6H), 2.44 (s, 3H), 2.17–2.36 (m, 6H) ppm. ^13^C-NMR (400 MHz, DMSO-*d*_6_) δ: 169.1, 164.3, 151.1, 144.2, 138.0, 131.8, 127.8, 126.7, 126.0, 122.2, 121.2, 112.5, 111.1, 57.2, 53.7, 52.0, 35.9, 25.3, 23.7, 13.4, 10.6, 7.2. MS-ESI (*m*/*z*): 472.6 (M + H)^+^, 474.6 (M + H)^+^. 

*(Z)-5-[(5-Bromo-2-oxo-1,2-dihydro-3H-pyrrolo[2,3-b]pyridin-3-ylidene)methyl]-2,4-dimethyl-N-[2-(4-methylpiperazin-1-yl)ethyl]-1H-pyrrole-3-carboxamide* (**23d**): Yield: 32%. m.p.: 244–246 °C. ^1^H-NMR (500 MHz, DMSO-*d*_6_) δ: 13.24 (s, 1H), 9.48 (s, 1H), 8.14 (d, *J =* 2.0 Hz, 1H), 7.77 (d, *J =* 2.0 Hz, 1H), 7.26 (s, 1H), 6.54 (t, *J =* 5.5 Hz, 1H), 3.55–3.58 (m, 2H), 2.64–2.66 (m, 4H), 2.58 (s, 3H), 2.43–2.50 (m, 4H), 2.37 (s, 3H), 2.30 (s, 3H), 1.60–1.64 (m, 2H) ppm. ^13^C-NMR (400 MHz, DMSO-*d*_6_) δ: 169.1, 164.2, 151.1, 144.1, 137.9, 131.7, 127.7, 126.7, 126.0, 122.2, 121.3, 112.5, 111.0, 56.7, 54.8, 52.5, 45.8, 36.1, 13.3, 10.6. MS-ESI (*m*/*z*): 487.7 (M + H)^+^, 489.7 (M + H)^+^. 

*(S,Z)-5-[(5-Bromo-2-oxo-1,2-dihydro-3H-pyrrolo[2,3-b]pyridin-3-ylidene)methyl]-N-(2-hydroxy-3-morpholinopropyl)-2,4-dimethyl-1H-pyrrole-3-carboxamide* (**23e**): Yield: 25%. m.p.: 284–286 °C. ^1^H-NMR (500 MHz, DMSO-*d*_6_) δ: 13.45 (s, 1H), 11.64 (s, 1H), 8.48 (d, *J =* 2.0 Hz, 1H), 8.10 (d, *J =* 2.0 Hz, 1H), 7.86 (s, 1H), 7.61 (t, *J =* 5.5 Hz, 1H), 4.76 (s, 1H), 3.78–3.80 (m, 1H), 3.55–3.59 (m, 4H), 3.13–3.18 (m, 2H), 2.46 (s, 3H), 2.44 (s, 3H), 2.27–2.36 (m, 6H) ppm. ^13^C-NMR (400 MHz, DMSO-d_6_) δ: 169.1, 164.6, 151.1, 144.2, 137.9, 131.9, 127.7, 126.7, 126.0, 122.2, 121.3, 112.5, 111.0, 66.7, 66.2, 63.0, 54.0, 43.9, 13.4, 10.6 ppm. MS-ESI (*m*/*z*): 504.6 (M + H)^+^, 506.6 (M + H)^+^. 

*(Z)-5-[(5-Bromo-2-oxo-1,2-dihydro-3H-pyrrolo[2,3-b]pyridin-3-ylidene)methyl]-2,4-dimethyl-N-[3-(piperidin-1-yl)propyl]-1H-pyrrole-3-carboxamide* (**23f**): Yield: 27%. m.p.: 236–238 °C. ^1^H-NMR (500 MHz, DMSO-*d*_6_) δ: 13.44 (s, 1H), 11.64 (s, 1H), 8.47 (d, *J =* 2.0 Hz, 1H), 8.10 (d, *J =* 2.0 Hz, 1H), 7.85 (s, 1H), 7.56 (t, *J =* 6.5 Hz, 1H), 3.24 (q, *J =* 12.5 Hz, 2H), 2.44 (s, 3H), 2.42 (s, 3H), 2.17–2.37 (m, 6H), 1.38–1.67 (m, 8H) ppm. ^13^C-NMR (400 MHz, DMSO-*d*_6_) δ: 169.1, 164.4, 151.1, 144.1, 137.7, 131.8, 127.7, 126.7, 126.0, 122.2, 121.6, 112.5, 111.0, 56.3, 54.0, 37.3, 26.5, 25.4, 24.0, 13.3, 10.5 ppm. MS-ESI (*m*/*z*): 486.0 (M + H)^+^, 488.0 (M + H)^+^.

*(Z)-5-[(5-Bromo-2-oxo-1,2-dihydro-3H-pyrrolo[2,3-b]pyridin-3-ylidene)methyl]-2,4-dimethyl-N-[4-(piperidin-1-yl)butyl]-1H-pyrrole-3-carboxamide* (**23g**): Yield: 33%. m.p.: 238–240 °C. ^1^H-NMR (500 MHz, DMSO-*d*_6_) δ: 13.44 (s, 1H), 11.64 (s, 1H), 8.47 (d, *J =* 2.0 Hz, 1H), 8.10 (d, *J =* 2.0 Hz, 1H), 7.85 (s, 1H), 7.73 (t, *J =* 5.5 Hz, 1H), 3.22 (q, *J =* 12.0 Hz, 2H), 2.43 (s, 3H), 2.41 (s, 3H), 2.22–2.37 (m, 6H), 1.43–1.53(m, 8H), 1.33–1.40 (m, 2H) ppm. ^13^C-NMR (400 MHz, DMSO-*d*_6_) δ: 169.1, 164.4, 151.1, 144.1, 137.6, 131.8, 127.7, 126.7, 126.0, 122.2, 121.8, 112.5, 110.9, 58.2, 54.0, 38.6, 27.3, 25.4, 24.1, 23.8, 13.2, 10.4 ppm. MS-ESI (*m*/*z*): 500.3 (M + H)^+^, 502.3 (M + H)^+^. 

*(Z)-5-[(5-Bromo-2-oxo-1,2-dihydro-3H-pyrrolo[2,3-b]pyridin-3-ylidene)methyl]-2,4-dimethyl-N-[3-(4-methylpiperazin-1-yl)propyl]-1H-pyrrole-3-carboxamide* (**23h**): Yield: 30%. m.p.: 238–240 °C. ^1^H-NMR (500 MHz, DMSO-*d*_6_) δ: 13.44 (s, 1H), 11.62 (s, 1H), 8.47 (d, *J =* 2.0 Hz, 1H), 8.10 (d, *J =* 2.0 Hz, 1H), 7.85 (s, 1H), 7.71 (t, *J =* 5.5 Hz, 1H), 3.22 (q, *J =* 12.5 Hz, 2H), 2.44 (s, 3H), 2.42 (s, 3H), 2.22–2.35 (m, 10H), 2.13 (s, 3H), 1.62–1.68 (m, 2H) ppm. ^13^C-NMR (400 MHz, DMSO-*d*_6_) δ: 169.1, 164.4, 151.1, 144.2, 137.7, 131.8, 127.8, 126.8, 126.0, 122.3, 121.7, 112.6, 111.0, 55.7, 54.7, 52.7, 45.7, 37.3, 26.6, 13.3, 10.5 ppm. MS-ESI (*m*/*z*): 501.3 (M + H)^+^, 503.3 (M + H)^+^.

*(Z)-5-[(5-Bromo-2-oxo-1,2-dihydro-3H-pyrrolo[2,3-b]pyridin-3-ylidene)methyl]-2,4-dimethyl-N-[4-(4-methylpiperazin-1-yl)butyl]-1H-pyrrole-3-carboxamide* (**23i**): Yield: 31%. m.p.: 238–240 °C. ^1^H-NMR (500 MHz, DMSO-*d*_6_) δ: 13.43 (s, 1H), 11.63 (s, 1H), 8.47 (d, *J =* 2.0 Hz, 1H), 8.10 (d, *J =* 2.0 Hz, 1H), 7.85 (s, 1H), 7.73 (t, *J =* 5.5 Hz, 1H), 3.22 (q, *J =* 12.0 Hz, 2H), 2.43 (s, 3H), 2.41 (s, 3H), 2.16–2.37 (m, 10H), 2.13 (s, 3H), 1.45–1.50 (m, 4H) ppm. ^13^C-NMR (400 MHz, DMSO-*d*_6_) δ: 169.1, 164.4, 151.1, 144.1, 137.6, 131.8, 127.7, 126.8, 126.0, 122.3, 121.8, 112.5, 110.9, 57.6, 54.8, 52.7, 45.7, 38.6, 27.3, 23.9, 13.3, 10.5 ppm. MS-ESI (*m*/*z*): 515.3 (M + H)^+^, 517.3 (M + H)^+^.

*(Z)-5-[(5-Bromo-2-oxo-1,2-dihydro-3H-pyrrolo[2,3-b]pyridin-3-ylidene)methyl]-N-[3-(dimethylamino)propyl]-2,4-dimethyl-1H-pyrrole-3-carboxamide* (**23j**): Yield: 29%. m.p.: 258–260 °C. ^1^H-NMR (500 MHz, DMSO-*d*_6_) δ: 13.44 (s, 1H), 11.61 (s, 1H), 8.47 (d, *J =* 2.0 Hz, 1H), 8.10 (d, *J =* 2.0 Hz, 1H), 7.85 (s, 1H), 7.73 (t, *J =* 5.5 Hz, 1H), 3.25 (q, *J =* 12.5 Hz, 2H), 2.45 (s, 3H), 2.42 (s, 3H), 2.30 (t, *J =* 6.5 Hz, 2H), 2.16 (s, 6H), 1.62–1.68 (m, 2H) ppm. ^13^C-NMR (400 MHz, DMSO-*d*_6_) δ: 169.1, 164.3, 151.1, 144.1, 137.8, 131.8, 127.7, 126.7, 126.0, 122.2, 121.5, 112.5, 111.0, 57.1, 45.2, 37.3, 27.1, 13.3, 10.5 ppm. MS-ESI (*m*/*z*): 446.2 (M + H)^+^, 448.2 (M + H)^+^. 

*(Z)-5-[(5-Bromo-2-oxo-1,2-dihydro-3H-pyrrolo[2,3-b]pyridin-3-ylidene)methyl]-N-[3-(diethylamino)propyl]-2,4-dimethyl-1H-pyrrole-3-carboxamide* (**23k**): Yield: 30%. m.p.: 234–236 °C. ^1^H-NMR (500 MHz, DMSO-*d*_6_) δ: 13.45 (s, 1H), 11.63 (s, 1H), 8.47 (d, *J =* 2.0 Hz, 1H), 8.11 (d, *J =* 2.0 Hz, 1H), 7.86 (s, 1H), 7.74 (t, *J =* 5.5 Hz, 1H), 3.25–3.31 (m, 2H), 3.06–3.15 (m, 6H), 2.47 (s, 3H), 2.44 (s, 3H), 1.86–1.92 (m, 2H), 1.21 (t, *J =* 7.2 Hz, 6H) ppm. ^13^C-NMR (400 MHz, DMSO-*d*_6_) δ: 169.1, 164.8, 151.2, 144.2, 137.9, 131.8, 127.8, 126.8, 126.0, 122.2, 121.1, 112.5, 111.2, 48.4, 46.0, 36.0, 23.5, 13.4, 10.6, 8.4 ppm. MS-ESI (*m*/*z*): 474.3 (M + H)^+^, 476.3 (M + H)^+^.

*5-[(Z)-(5-Bromo-2-oxo-1,2-dihydro-3H-pyrrolo[2,3-b]pyridin-3-ylidene)methyl]-N-{3-[(E)-3-(methoxyimino)pyrrolidin-1-yl]propyl}-2,4-dimethyl-1H-pyrrole-3-carboxamide* (**23l**): Yield: 27%. m.p.: 226–228 °C. ^1^H-NMR (500 MHz, DMSO-*d*_6_) δ: 13.45 (s, 1H), 11.63 (s, 1H), 8.47 (d, *J =* 2.0 Hz, 1H), 8.10 (d, *J =* 2.0 Hz, 1H), 7.85 (s, 1H), 7.75 (t, *J =* 5.5 Hz, 1H), 3.95–4.10 (m, 4H), 3.78 (s, 3H), 3.48–3.56 (m, 2H), 3.28–3.32 (m, 2H), 2.63–2.69 (m, 2H), 2.44 (s, 3H), 2.42 (s, 3H), 1.80–1.85 (m, 2H) ppm. ^13^C-NMR (400 MHz, DMSO-*d*_6_) δ: 169.1, 164.5, 154.1, 151.1, 144.2, 137.7, 131.8, 127.7, 126.7, 126.0, 122.2, 121.4, 112.5, 111.0, 62.7, 61.4, 45.6, 43.8, 35.6, 28.9, 18.5, 13.3, 10.5 ppm. MS-ESI (*m*/*z*): 515.1 (M + H)^+^, 517.1 (M + H)^+^.

*5-[(Z)-(5-Bromo-2-oxo-1,2-dihydro-3H-pyrrolo[2,3-b]pyridin-3-ylidene)methyl]-N-{2-[(E)-3-(methoxyimino)piperidin-1-yl]ethyl}-2,4-dimethyl-1H-pyrrole-3-carboxamide* (**23m**): Yield: 26%. m.p.: 198–200 °C. ^1^H-NMR (500 MHz, DMSO-*d*_6_) δ: 13.45 (s, 1H), 11.61 (s, 1H), 8.47 (d, *J =* 2.0 Hz, 1H), 8.10 (d, *J =* 2.0 Hz, 1H), 7.85 (s, 1H), 7.80 (t, *J =* 5.5 Hz, 1H), 3.98–4.24 (m, 4H), 3.74 (s, 3H), 3.46–3.48 (m, 4H), 2.44 (s, 3H), 2.42 (s, 3H), 2.22–2.36 (m, 2H), 1.63–1.69 (m, 2H) ppm. ^13^C-NMR (400 MHz, DMSO-*d*_6_) δ: 169.1, 164.6, 153.9, 151.1, 144.2, 137.8, 131.9, 127.8, 126.8, 126.0, 122.2, 121.2, 112.5, 111.1, 63.8, 61.0, 56.0, 43.3, 38.2, 22.8, 18.5, 13.3, 10.4 ppm. MS-ESI (*m*/*z*): 515.3 (M + H)^+^, 517.3 (M + H)^+^. 

*(Z)-5-[(5-Bromo-2-oxo-1,2-dihydro-3H-pyrrolo[2,3-b]pyridin-3-ylidene)methyl]-N-{2-[4-(methoxyimino)piperidin-1-yl]ethyl}-2,4-dimethyl-1H-pyrrole-3-carboxamide* (**23n**): Yield: 29%. m.p.: 262–264 °C. ^1^H-NMR (500 MHz, DMSO-*d*_6_) δ: 13.44 (s, 1H), 11.62 (s, 1H), 8.46 (d, *J =* 2.0 Hz, 1H), 8.10 (d, *J =* 2.0 Hz, 1H), 7.85 (s, 1H), 7.58 (t, *J =* 5.5 Hz, 1H), 3.71 (s, 3H), 3.33–3.39 (m, 2H), 2.56 (t, *J =* 6.0 Hz, 2H), 2.47–2.52 (m, 6H), 2.46 (s, 3H), 2.44 (s, 3H), 2.23 (t, *J =* 6.0 Hz, 2H) ppm. ^13^C-NMR (400 MHz, DMSO-*d*_6_) δ: 169.1, 164.2, 156.3, 151.1, 144.2, 137.9, 131.8, 127.7, 126.7, 126.0, 122.2, 121.4, 112.5, 111.0, 60.6, 56.2, 53.1, 51.7, 36.4, 30.8, 24.9, 13.3, 10.5 ppm. MS-ESI (*m*/*z*): 515.2 (M + H)^+^, 517.3 (M + H)^+^.

*(Z)-5-[(5-Bromo-2-oxo-1,2-dihydro-3H-pyrrolo[2,3-b]pyridin-3-ylidene)methyl]-N-{3-[4-(methoxyimino)piperidin-1-yl]propyl}-2,4-dimethyl-1H-pyrrole-3-carboxamide* (**23o**): Yield: 32%. m.p.: 233–235 °C. ^1^H-NMR (500 MHz, DMSO-*d*_6_) δ: 13.44 (s, 1H), 11.63 (s, 1H), 8.47 (d, *J =* 2.0 Hz, 1H), 8.10 (d, *J =* 2.0 Hz, 1H), 7.85 (s, 1H), 7.71 (t, *J =* 5.5 Hz, 1H), 3.71 (s, 3H), 3.25 (q, *J =* 12.5 Hz, 2H), 2.45–2.49 (m, 6H), 2.44 (s, 3H), 2.42 (s, 3H), 2.38 (t, *J =* 5.9 Hz, 2H), 2.22 (t, *J =* 7.0 Hz, 2H), 1.65–1.71 (m, 2H) ppm. ^13^C-NMR (400 MHz, DMSO-*d*_6_) δ: 169.1, 164.4, 156.4, 151.1, 144.2, 137.7, 131.8, 127.7, 126.8, 126.0, 122.2, 121.6, 112.5, 111.0, 60.5, 55.0, 53.3, 52.0, 37.2, 30.7, 26.9, 24.8, 13.3, 10.5 ppm. MS-ESI (*m*/*z*): 529.3 (M + H)^+^, 531.3 (M + H)^+^.

*(Z)-5-[(5-Bromo-2-oxo-1,2-dihydro-3H-pyrrolo[2,3-b]pyridin-3-ylidene)methyl]-N-{3-[4-(ethoxyimino)piperidin-1-yl]propyl}-2,4-dimethyl-1H-pyrrole-3-carboxamide* (**23p**): Yield: 31%. m.p.: 237–239 °C. ^1^H-NMR (500 MHz, DMSO-*d*_6_) δ: 13.44 (s, 1H), 11.63 (s, 1H), 8.47 (d, *J =* 2.0 Hz, 1H), 8.10 (d, *J =* 2.0 Hz, 1H), 7.85 (s, 1H), 7.71 (t, *J =* 5.5 Hz, 1H), 3.97 (q, *J =* 7.0 Hz, 2H), 3.25 (q, *J =* 12.5 Hz, 2H), 2.45–2.48 (m, 6H), 2.44 (s, 3H), 2.42 (s, 3H), 2.37–2.40 (m, 2H), 2.22–2.24 (m, 2H), 1.65–1.71 (m, 2H), 1.16 (t, *J =* 7.0 Hz, 3H) ppm. ^13^C-NMR (400 MHz, DMSO-*d*_6_) δ: 169.1, 164.4, 156.0, 151.1, 144.1, 137.7, 131.8, 127.7, 126.7, 126.0, 122.2, 121.6, 112.5, 111.0, 67.8, 55.0, 53.4, 52.0, 37.2, 30.8, 26.9, 24.9, 14.5, 13.3, 10.5 ppm. MS-ESI (*m*/*z*): 543.3 (M + H)^+^, 545.3 (M + H)^+^. 

*(Z)-N-{3-[4-(Benzyloxyimino)piperidin-1-yl]propyl}-5-[(5-bromo-2-oxo-1,2-dihydro-3H-pyrrolo[2,3-b]pyridin-3-ylidene)methyl)]-2,4-dimethyl-1H-pyrrole-3-carboxamide* (**23q**): Yield: 30%. m.p.: 226–228 °C. ^1^H-NMR (500 MHz, DMSO-*d*_6_) δ: 13.44 (s, 1H), 11.63 (s, 1H), 8.47 (d, *J =* 2.0 Hz, 1H), 8.10 (d, *J =* 2.0 Hz, 1H), 7.85 (s, 1H), 7.71 (t, *J =* 5.5 Hz, 1H), 7.27–7.36 (m, 5H), 4.99 (s, 2H), 3.25 (q, *J =* 12.5 Hz, 2H), 2.51–2.53 (m, 2H), 2.47–2.48 (m, 4H), 2.44 (s, 3H), 2.42 (s, 3H), 2.37–2.40 (m, 2H), 2.22 (t, *J =* 5.8 Hz, 2H), 1.65–1.71 (m, 2H) ppm. ^13^C-NMR (400 MHz, DMSO-*d*_6_) δ: 169.1, 164.4, 157.2, 151.1, 144.1, 138.2, 137.7, 131.8, 128.2, 127.7, 127.5, 126.7, 126.0, 122.2, 121.6, 112.5, 111.0, 74.3, 55.0, 53.3, 52.0, 37.2, 30.8, 26.9, 25.0, 13.3, 10.5 ppm. MS-ESI (*m*/*z*): 605.3(M + H)^+^, 607.3 (M + H)^+^.

## 4. Conclusions

In summary, a series of novel 5-bromo-7-azaindolin-2-one derivatives containing a 2,4-dimethyl-1*H*-pyrrole-3-carboxamide moiety were designed, synthesized and evaluated for their in vitro antitumor activity by MTT assay. Our results reveal that many target compounds exhibit broad-spectrum antitumor potency which is better than Sunitinib. The most active compound **23p** (IC_50_: 2.357–3.012 μM) was found 11.3- to 8.4-fold more potent than Sunitinib against all of the tested cell lines, HepG2, A549 and Skov-3, respectively. Studies to determine the in vivo pharmacokinetics and efficacy of compounds **23c**, **23d** and **23p** against some selected tumor cell lines are currently underway.

## Figures and Tables

**Table 1:**
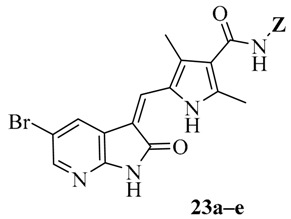
In vitro activity of target compounds **23a**–**e** against six cell lines.

Compound	Z	% Inhibition ^a^	IC50 (μM)					
			MCF-7	HepG2	HT-29	A549	PANC-1	SKOV-3
**23a**	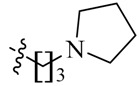	87.29	12.790	7.060	8.893	4.993	14.132	5.766
**23b**	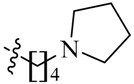	83.61	27.457	6.852	10.820	6.555	13.672	5.978
**23c**	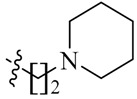	81.46	17.946	7.882	9.524	3.103	9.410	6.669
**23d**	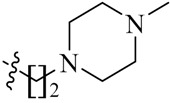	84.98	15.052	6.316	10.388	6.250	9.512	3.721
**23e**	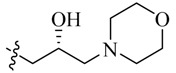	46.83	65.054	36.964	19.642	26.031	33.314	29.685
Sunitinib		27.79	65.606	41.805	31.774	29.257	54.916	31.985

^a^ % inhibition of **23a**–**e** and Sunitinib against MCF-7 (at 30 μM).

**Table 2:**
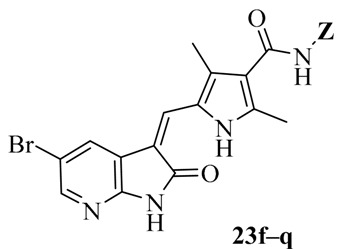
In vitro activity of target compounds **23f**–**q** against three cell lines.

Compound	Z	IC_50_(μM)		
		HepG2	A549	SKOV-3
**23f**	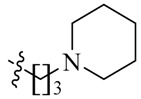	7.144	6.982	5.023
**23g**	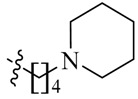	7.803	6.137	7.507
**23h**	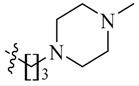	11.543	13.506	15.126
**23i**	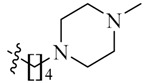	7.657	6.254	6.470
**23j**	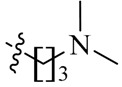	14.916	13.443	14.916
**23k**	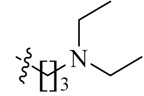	12.229	10.273	10.998
**23l**	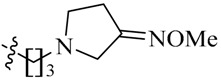	60.617	59.319	40.087
**23m**	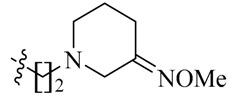	99.667	119.493	25.090
**23n**	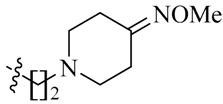	22.054	36.509	17.296
**23o**	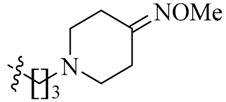	6.828	7.731	7.747
**23p**	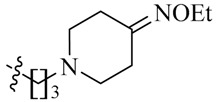	3.012	2.357	2.659
**23q**	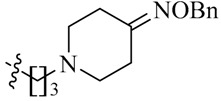	5.878	6.681	4.075
Sunitinib		33.999	31.594	49.036
